# Kir4.1 Dysfunction in the Pathophysiology of Depression: A Systematic Review

**DOI:** 10.3390/cells10102628

**Published:** 2021-10-01

**Authors:** Stefania Della Vecchia, Maria Marchese, Filippo Maria Santorelli, Federico Sicca

**Affiliations:** 1 Department of Developmental Neuroscience, IRCCS Stella Maris Foundation, Calambrone, 56128 Pisa, Italy; stefania.dellavecchia@fsm.unipi.it; 2 Department of Clinical and Experimental Medicine, University of Pisa, 56126 Pisa, Italy; 3 Department of Molecular Medicine, IRCCS Stella Maris Foundation, Via dei Giacinti 2, 56128 Pisa, Italy; maria.marchese@fsm.unipi.it; 4 Child Neuropsychiatric Unit, USL Centro Toscana, 59100 Prato, Italy

**Keywords:** Kir4.1, depression, astrocytes, glutamate, serotonin, BDNF

## Abstract

A serotonergic dysfunction has been largely postulated as the main cause of depression, mainly due to its effective response to drugs that increase the serotonergic tone, still currently the first therapeutic line in this mood disorder. However, other dysfunctional pathomechanisms are likely involved in the disorder, and this may in part explain why some individuals with depression are resistant to serotonergic therapies. Among these, emerging evidence suggests a role for the astrocytic inward rectifier potassium channel 4.1 (Kir4.1) as an important modulator of neuronal excitability and glutamate metabolism. To discuss the relationship between Kir4.1 dysfunction and depression, a systematic review was performed according to the PRISMA statement. Searches were conducted across PubMed, Scopus, and Web of Science by two independent reviewers. Twelve studies met the inclusion criteria, analyzing Kir4.1 relationships with depression, through in vitro, in vivo, and *post-mortem* investigations. Increasing, yet not conclusive, evidence suggests a potential pathogenic role for Kir4.1 upregulation in depression. However, the actual contribution in the diverse subtypes of the disorder and in the comorbid conditions, for example, the epilepsy-depression comorbidity, remain elusive. Further studies are needed to better define the clinical phenotype associated with Kir4.1 dysfunction in humans and the molecular mechanisms by which it contributes to depression and implications for future treatments.

## 1. Introduction

Depression is a common and serious mood disorder, occurring at any age, from childhood to late adulthood [1,2], and presenting in multiple clinical forms [3]. Its neurobiology is only partially known and is thought to rely on a series of different and in part concurrent mechanisms that underlie both the core psychopathology of the disorder and the heterogeneous clinical variants characterizing its presentation [4].

The initial “Monoaminergic hypothesis” [5,6], later further revised as a “Serotonin (5-HT) hypothesis” [7,8], historically relies on the clinical evidence of the depressogenic effect of reserpine [9,10], a drug that depletes brain monoamines by disrupting their vesicular storage. The long-lasting clinical experience with antidepressant drugs, such as monoamine inhibitors, tricyclic antidepressants (TCAs), selective serotonin and serotonin/norepinephrine reuptake inhibitors (respectively, SSRIs and SNRIs) [11,12], which are all known to relieve depressive symptoms mainly through an increase in serotonin levels at the synaptic cleft [13], has further emphasized the crucial role of serotonergic dysfunction in the disorder. Indeed, currently, clinical evidence and relevant guidelines still support the use of SSRIs as the first-line drugs in depression, in both adults [12] and young people [14]. The “serotonin hypothesis”, however, has become too simplistic over time and some criticisms have questioned the quasi-exclusive role of serotonin in the disorder. These criticisms mainly result from the finding of a wide response to conventional antidepressant treatments, with 10–30% of patients displaying drug-refractoriness [15]. Moreover, the discrepancy between the rapid increase in synaptic monoamine levels after antidepressant administration [13], and the late clinical response (typically, 2–6 weeks after starting treatment) seen in treated patients [16,17], further weakens the serotonergic hypothesis as the exclusive mechanism in the disorder.

In the 1990s, the discovery of the rapid antidepressant properties of ketamine, an N-methyl-D-aspartate receptor (NMDAR) antagonist, has strongly suggested a role for glutamate in the pathogenesis of depression [18,19], emphasizing its participation in neuroplasticity [20]. Neurotrophins, particularly the brain-derived neuronal growth factor (BDNF) [21,22], are also thought to play a pivotal role in the pathophysiology of depression; a reduction of the BDNF-related trophic support, indeed, seems to entail neuronal and glial atrophy in brain areas involved in the disorder (hippocampus, amygdala, prefrontal cortex) [23]. In the same areas, the levels of neurotrophins increase under antidepressants administration in a period of 2–6 weeks, possibly explaining their contribution to symptoms reversion and the previously discussed time-lag between treatment starting and clinical response [16].

Several reviews have, finally, focused on the role of astrocytes in depression pathogenesis, leading to a significant shift from a neuro-centric approach toward an astrocyte-centric vision of the disorder [23,24,25]. Growing evidence is showing that astrocytes, the most numerous cells in the mammalian brain, strongly influence brain function through modulation of metabolic and neurotransmission activities at the tripartite synapse [26,27], thus playing a role in many neurologic and psychiatric disorders [28,29,30]. Interestingly, *post-mortem* studies on patients diagnosed with major depressive disorders [24,25,31,32,33,34] and animal models [24,35] have shown quantitative and morphological abnormalities in brain regions (i.e., fronto-limbic areas) traditionally associated with depression. Astrocyte dysfunction is supposed to contribute to the pathogenesis of depression through several mechanisms, i.e., by affecting the monoaminergic system, modulating neuronal activity, or altering the excitatory-inhibitory balance, and by disturbing the neurotrophic support of neuronal networks [23]. Among the large number of astrocyte proteins acting at the tripartite synapse, recent evidence has focused on the inward rectifier potassium channel 4.1 (Kir4.1 channel; *KCNJ10* gene) as a possible contributor to several neuropsychiatric diseases [36], including depression [37,38,39,40,41]. The inwardly rectifying role of Kir4.1, indeed, helps maintaining the ionic and osmotic environment in the extracellular space, the so-called spatial K^+^ buffering [42]. The polarized transport of K^+^ in astrocytes is essential for normal neuronal activity and excitability and for synaptic functions [36,43]. Interestingly, several antidepressant drugs have been shown to affect Kir4.1 function or trafficking [44,45,46], and an altered expression of the Kir4.1 channel in different brain areas has been associated with depression-like behaviors in rodents [37,47] and observed in brain from patients with depression in *post-mortem* studies [38,39].

In this study, we have systematically reviewed the literature evidence on this topic to further clarify the relationship between Kir4.1 function and the antidepressant drugs that are used to treat human depression, and the role of the channel in the neurobiology of the disorder.

## 2. Methods

The study was conducted according to the Preferred Reporting Items for Systematic Reviews and Meta-Analyses (PRISMA) guidelines. A systematic search strategy was carried out in PubMed, Scopus, and Web of Science databases from their inception until June 2021. We also searched in papers’ reference lists to identify additional studies meeting our criteria. Free text terms have been combined as follows for all databases: (depress* OR antidepre*) AND (Kir4.1 OR inward rectifying 4.1 OR KCNJ10). We used the research tool “Zotero” (https://www.zotero.org/download/ (accessed on 7 June 2021)) to collect all the results in a single library. The full text of all potentially eligible articles and their supplementary information were obtained and independently assessed by two authors (S.D.V. and M.M.). We resolved any ambiguities about eligibility through discussion. Studies were included if they reported on the relationship between the astrocytic Kir4.1 channel and (1) antidepressant drugs or (2) depression. The antidepressant drugs that were considered for the inclusion of the studies in our systematic review were those recommended in the treatment guidelines for adults [12] and young people [14] (i.e., SSRIs, SNRIs, TCAs, monoamine oxidase inhibitors, atypical antidepressants), and the more recently FDA (Food and Drug Administration) approved drug for resistant depression ketamine [48].

The selection excluded: (a) duplicates; (b) studies not concerning the aim of the paper; (c) studies focusing on drugs that were not approved for depression treatment in humans or with alleged antidepressant effects only based on limited evidence in animal models; (d) articles written in a language other than English, French, or Italian; (e) reviews, meeting abstracts and editorial comments.

The search strategy for the systematic review can be found in the Supplementary Materials.

## 3. Results

**Figure 1:**
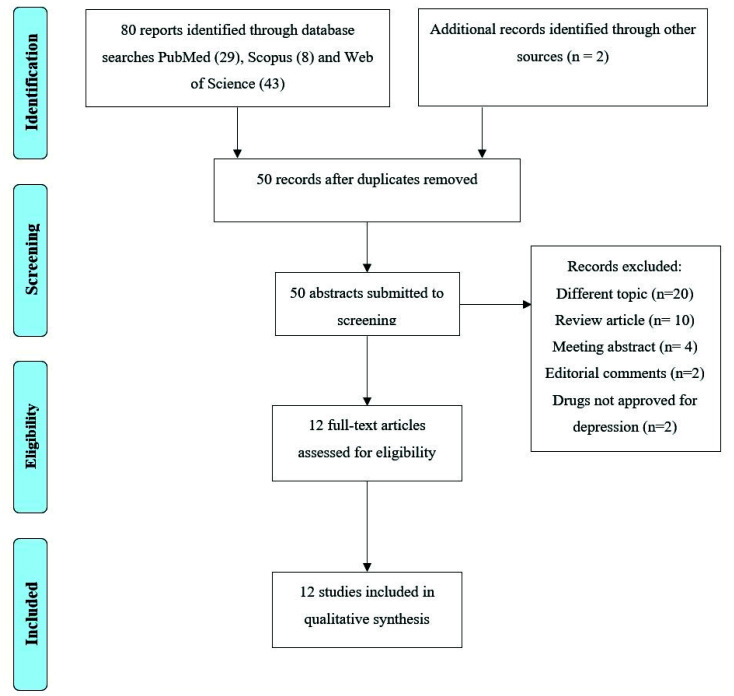
PRISMA flow diagram of search yield, screening, and inclusion steps.

The PRISMA Flow Diagram [49] of the review process is presented in Figure 1. The search of PubMed, Scopus and Web of Science databases provided a total of 80 records. Two additional studies were selected by checking the references of the identified relevant papers. After adjusting for duplicates, 50 records remained. Of these, 20 papers were excluded because they dealt with a different topic, 16 because they were reviews, meeting abstracts, or editorial comments, and 2 further studies because they reported on molecules with alleged antidepressant effects based only on preclinical studies. A total of 12 studies were therefore identified for inclusion in the review [37,38,39,44,45,46,50,51,52,53,54,55]. We classified these studies according to their focus on (1) Kir4.1 and depression (2) Kir4.1 and antidepressants. Table 1 and Table 2 summarize the main results of the studies included in this work.

### 3.1. Kir4.1 Channel Expression and Depression

Studies on patients and animal models highlighted an alteration of astrocytic Kir4.1 channel expression in a variety of central nervous system (CNS) pathological conditions [43]. Among them, an altered expression of these astrocytic channels has also been found in depression, both in rodent models [37,54] and in *post-mortem* human studies [38,39].

#### 3.1.1. Down-Regulation of the Astrocytic Kir4.1 Channel

Following a set of separate evidence pointing towards a pathogenic role for astrocytes in major depressive disorder (MDD), Medina and colleagues investigated the glial syncytial function in *post-mortem* hippocampal samples from 13 individuals with MDD diagnosis and 10 controls [38]. Hippocampal tissues were electively used for the experiments due to the important roles of this limbic structure not only for learning and memory but also for emotional behaviors [56,57] and its involvement in the pathophysiology of depression [58,59]. The investigation of hippocampal gene expression revealed 1114 genes differentially expressed between control and MDD groups. A search focused on glial function showed a downregulation, in the hippocampus of MDD patients, of many genes involved in astrocyte metabolism. These included the *KCNJ10* gene, coding for Kir4.1, *AQP4,* which is a member of the aquaporin family important for water homeostasis and K^+^ buffering [60], *SLC1A2* and *SLC1A3* involved in glutamate reuptake function, and *GJA1*, a gap junctions’ component that participates in the formation of astrocyte syncytium. Although *KCNJ10* mRNA displayed a statistically significant reduction in MDD samples on microarray analyses, the authors could not establish whether the decreased expression of *KCNJ10* was a cause or consequence of the depressive condition.

#### 3.1.2. Up-Regulation of Astrocytic Kir4.1 Channel

Very interesting insights have emerged from two studies published simultaneously by the same research group [37,54]. Their research follows previous evidence from animal and human studies demonstrating that dysfunctional activity of the lateral habenula (LHb), an epithalamic relay nucleus connecting forebrain limbic structures with midbrain monoaminergic centers and primarily hosting glutamatergic neurons, is involved in the development of depressive features [61,62,63]. Using the “Congenitally Learned Helpless Rat” (cLH), an established animal model for congenital depression, Yang and colleagues [54] found that the systemic injection and the local infusion into the rat LHb of ketamine, an NMDAR antagonist, equally result in the reversion of depressive symptoms. This finding likely suggests a specific role for the LHb in mediating the antidepressant effect of ketamine-induced NMDAR blockade. To confirm the role of the LHb neuronal hyperexcitability in depression, the authors performed electrophysiological recordings in the LHb neurons of two rodent models of depression (the cLH rat and the chronic restraint stress (CRS) mouse) and observed a significant increase in bursting activity compared with control animals. By applying optogenetic tools driving the bursting activity in the LHb, they also demonstrated that the increased bursts of mice LHb neurons is a sufficient mechanism underlying aversion and depression-like symptoms. Then, they demonstrated that the excessive bursting of LHb neurons is triggered by hyperpolarization of neurons and found that NMDA receptors and T-type sensitive calcium channels (T-VSCCs) are crucial for LHb bursting activity. Moreover, the addition of ketamine and mibefradil (a T-VSCC blocker) to LHb brain slices eliminates and reduces the bursting activity of LHb neurons, respectively, but this phenomenon does not affect the resting membrane potential. Thus, in addition to reversing depressive symptoms, ketamine injection reduced LHb bursting activity in both cLH rats and CRS mice. The same rapid antidepressant effect was achieved by blocking the T-VSCC channels through systemic injection of the antiepileptic drug ethosuximide or by bilateral infusion of mibefradil into the LHb of CRS mice. Thus, the authors suggested that increased bursting activity in LHb neurons drives depression, and that the rapid antidepressant effect of ketamine might be related to the quenching of LHb neurons.

In their accompanying paper, Cui and colleagues [37] also investigated the possible role of astrocytes in depression, focusing their study on the astrocytic Kir4.1 channel, by examining if it had a role in regulating the resting membrane potential (RMP) and bursting activity of LHb neurons. The authors assessed the localization and expression of Kir4.1 channels in the LHb of two animal models of depression (the already investigated cLH rat and the lipopolysaccharide (LPS)-treated rat), demonstrating that Kir4.1 is expressed both on astrocytic endfeet surrounding synapses and on astrocytic processes contacting the neuronal soma, and showing an up-regulation of Kir4.1 in the LHb of both animal models, at least in part dependent on transcription. This upregulation, moreover, occurred 60–90 days after birth, just when the rodents showed their first depression-like symptoms. The synchrony between the increased expression of Kir4.1 in the LHb and the depressive phenotype suggested a causal role for Kir4.1 upregulation in depression. This was further corroborated by linking depression-like behaviors and overexpression of adeno-associated virus (AAV) mRNA/*KCNJ10* in the LHb of wild-type mice. These results suggested that increased extracellular potassium clearance mediated by Kir4.1 overexpression might underlie the neuronal hyperpolarization required for burst initiation in LHb. Moreover, downregulation of Kir4.1 in the LHb of cLH rats, through short hairpin RNAs (shRNAs) or dominant-negative constructs that block its function, caused a reduction in bursting activity and the rescue of depressive behaviors. To explain the cascade mechanisms by which Kir4.1 overexpression led to increased bursting of the LHb neurons and ultimately to depressive symptoms, the authors postulated the hypothesis that Kir4.1-mediated neuronal hyperpolarization could inactivate the T-VSCCs and induce the activation of the NMDA receptors, which triggered the LHb neuronal bursting. The excessive bursting of LHb neurons could inhibit the monoaminergic centers located downstream, activating the tegmental rostromedial nucleus. Monoamine deficiency thus would be responsible for the depressive phenotype.

Following the important evidence provided by these two studies, another study [39] demonstrated upregulation of Kir4.1 channels by western blotting in *post-mortem* parietal cortex of patients with MDD but not in brain areas from patients with different psychiatric disorders such as schizophrenia and bipolar disorder and in a non-affected control group.

#### 3.1.3. No Change of Expression of Kir4.1 Astrocytic Channel

In contrast to previous studies, one work [55] performed on CSDS (susceptible mice after chronic social defeat stress), a model of depression based on the stress paradigm [64], found no change in Kir4.1 expression in the prefrontal cortex [65,66], nucleus accumbens [67,68] and hippocampus [69] and concluded that Kir4.1 is not essential in the CSDS model of depression.

### 3.2. Kir4.1 Channels and Drugs with Antidepressant Action

#### 3.2.1. Kir4.1 Channels, TCAs, and SSRIs

The first association of astrocytic Kir4.1 channels with depression emerged from the evidence that several antidepressant drugs, in particular TCAs and SSRIs, are able to inhibit currents through the channel [44,45] by direct interaction [52], suggesting that Kir4.1 inhibition could have an antidepressant action [40]. The inhibition of Kir4.1 channels by antidepressants may induce neuronal excitability, since the resulting altered potassium siphoning would lead to an increased extracellular [K^+^] and reduced clearance of extracellular glutamate [40]. This mechanism could hypothetically also explain the pro-convulsive side effect of some antidepressant drugs [40].

In vitro studies performed on HEK293T cells transfected with human Kir4.1 complementary DNA (cDNA) have shown that TCAs (such as amitriptyline, nortriptyline, desipramine, and imipramine) inhibit Kir4.1 channel activity in a voltage-dependent manner [44]. Whole-cell patch-clamp recordings after administration of these drugs showed, indeed, an inhibition of both outward and inward potassium currents, with a reversible effect after elimination of the drug. The same research group [45] showed similar inhibitory effects on Kir4.1 currents, using selective serotonin reuptake inhibitors (SSRIs), i.e., sertraline, fluoxetine, and fluvoxamine, while the tetracyclic (mianserin) or the 5-HT1A receptor-related (buspirone) displayed no significant effects on channel function. Fluoxetine and sertraline displayed a stronger, voltage-independent effect, with respect to fluvoxamine, which instead inhibited Kir4.1 in a voltage-dependent manner.

Inhibition of Kir4.1 has been therefore postulated as a possible mechanism contributing to the antidepressant effect of tricyclic drugs, perhaps by enhancing neuronal excitability through the increase in extracellular potassium levels [40]. The pro-excitatory effect of Kir4.1 inhibition by antidepressants might also account, at least in part, for the reduced convulsive threshold induced by some of these drugs in humans [44,45]. Using chimeric and site directed mutants of Kir4.1 expressed in Xenopus Laevis oocytes and computational analyses of three-dimensional arrangements of the ligands, it has been suggested that the inhibitory effect of antidepressants (SSRIs and TCAs) on Kir4.1 can be due to a direct ionic interaction of the drugs with channels’ pore residues [52].

#### 3.2.2. Kir4.1 Channels, Antidepressants and BDNF

A study on primary mouse astrocyte cultures and on HEK293T cells has shown that inducing a loss of function of Kir4.1, by either antidepressant drugs (fluoxetine, sertraline, fluvoxamine, imipramine, and mianserin) or RNA silencing (siRNA), results in a significant increase in mRNA and protein levels of BDNF [53], a modulator of both depression [21,70] and epileptogenesis [71,72].

Recently, in vivo experiments have confirmed the possible link between downregulation of Kir4.1 and upregulation of the BDNF/TrkB pathway [50,51]. Two studies in adult rats [50,51] treated repeatedly with intraperitoneal injections of fluoxetine for 15 days showed neuroplastic changes in the medial prefrontal cortex, a brain region involved in the pathogenesis of depression and in the antidepressant action of Fluoxetine [73,74], likely due to both downregulation of Kir4.1 and upregulation of BDNF signaling. Therefore, the Kir4.1 downregulation and the BDNF/TrkB signaling pathway upregulation, the latter at least in part induced by Kir4.1 inhibition itself, could both contribute to the long-term neurotrophic effects of chronic fluoxetine treatment in rodent models.

#### 3.2.3. Kir4.1 Channels and Ketamine

Interesting insights have emerged from a study investigating the effect of ketamine, an NMDA receptor antagonist, used in clinical practice both as an anesthetic and as a rapid-acting antidepressant drug, on Kir4.1 metabolism [46]. Adding sub-anesthetic doses of ketamine to cultured rat cortex astrocytes resulted in reduced intracellular trafficking and lower plasmalemmal density of Kir4.1 channels. This finding suggests that the antidepressant effects of ketamine may rely, at least in part, on Kir4.1 functional modulation at astrocytes [46]. The molecular mechanisms underlying the rapid action of ketamine remain poorly understood, however, with potential explanations relating to the aforementioned downregulation of the Kir4.1 channels [46].

## 4. Discussion

**Figure 2:**
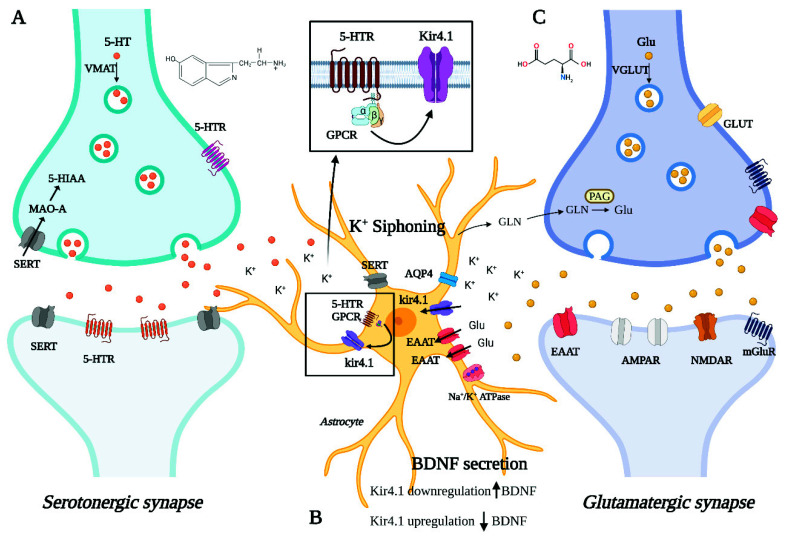
Graphical representation of a tripartite synapse where the following neurometabolic pathways involved in depression and possibly affected by Kir4.1 dysfunction are schematically illustrated: (**A**) Serotonergic transmission (**B**) astrocyte-mediated K^+^ siphoning, glutamate reuptake, and BDNF secretion (**C**) glutamatergic transmission.

In this systematic review, we have collected and investigated the literature data reporting on the role of the astrocytic Kir4.1 channel in the pathogenesis of depression. Our investigations support the hypothesis that Kir4.1 takes part in the complex mechanisms that underlie the disorder and influence its response to therapies. Our analyses of published work add further evidence that also astrocytes, and not only neurons, contribute to the neurobiology of psychiatric disorders [23,24]. Figure 2 provides a schematic representation of the tripartite synapse with the main pathways involved in the neurobiology of depression potentially affected by Kir4.1 dysfunction.

First, we focused on the possible consequences of channel up- and down-regulation on tripartite synapse metabolism, in particular on the astrocyte siphoning function and glutamatergic transmission, also highlighting the controversial findings that emerge from the most relevant recent literature. The mechanisms by which the altered channel expression may affect depression firstly concern the astrocyte-mediated potassium siphoning and the neuronal glutamate-uptake [37,75,76,77]. Both these metabolic processes may therefore contribute to depression development by impairing glutamatergic transmission. Given the prevalence of glutamatergic circuits in brain areas involved in mood disorders [20], their dysfunction is expected to be crucial for the development of the disorder [20,78]. Data in *post-mortem* tissues and in vivo lend further support to this mechanism. Additional evidence supporting a role for Kir4.1 overexpression in the disorder results from a recent work [47] where LPS mice (a rodent model for depression [79]) were treated with Ginsenoside Rg1, a molecule with known antidepressant properties in preclinical studies [80,81]. Interestingly, LPS mice displayed an increased hippocampal expression of Kir4.1, which was reduced by Ginsenoside Rg1 administration. In addition, the silencing of Kir4.1 in the hippocampus, after bilateral injection of shRNA, led to a significant improvement in depression-like behaviors contributing to rescue the pathological phenotype.

Second, we shifted the focus on other mechanisms, mainly serotonin- and neurotrophin-mediated, which likely interplay with Kir4.1 in depression pathogenesis. The studies described above underline a role of the astrocytic Kir4.1 channel in the neurobiology of depression that goes beyond the serotonergic system or influences it indirectly through the connection between the networks directly affected by Kir4.1 dysregulation and the downstream monoaminergic centers [37,54]. It has been recently shown that the application of 5-HT in mouse sensory cortex slices can induce the production of secondary messengers that act on Kir4.1 channels [82] to modify the extracellular potassium homeostasis and, thus, the neuronal activity. The evidence that serotonin can communicate with and modulate Kir4.1 activity adds further complexity to the bulk of pathomechanisms, underlying depression, where Kir4.1 may at the same time be an upstream and downstream interactor of serotonergic metabolites. Recent transcriptomic studies performed on rat raphe tissues have suggested that the ph-sensitivity of 5HT neurons mainly rely on the expression levels of the Kir5.1 channel, which are expressed in both serotonergic neurons and glial cells [83]. The role of the Kir5.1 channels in dorsal raphe (DR) may be relevant for depression since DR, as well as peri-coerulear 5-HT neurons, provide dense serotonergic projections to locus coeruleus (LC) neurons, which are, in turn, important for regulating fundamental behavioral functions such as cognition, anxiety, and mood [84,85]. On the other hand, Kir4.1 channels are only present in glial cells in raphe tissues; therefore, their altered expression is unlikely to affect the DR-LC network through direct mechanisms. However, we cannot rule out the possibility that an altered expression of Kir4.1 channels might affect the formation of Kir4.1/Kir5.1 heteromeric channels, as has been postulated to occur in vivo in cortical astrocytes [86,87] and in LC neurons [88].

In addition to serotonin, neurotrophins, particularly the neurotrophic factor derived from the brain (BDNF), are also involved in the neurobiology of depression [21,22,89,90]. Thus, another potential mechanism through which kir4.1 dysfunction could contribute to depression is by influencing astrocyte BDNF secretion [40,41]. Evidence that Kir4.1 inhibition increases astrocyte secretion of BDNF [53] led to the hypothesis that Kir4.1 overexpression may participate in the neurobiology of depression by reducing BDNF secretion [40,41]. However, targeted studies are needed to elucidate the effects of Kir4.1 dysfunction on serotonergic transmission and BDNF secretion.

The direct inhibition of Kir4.1 channels by administration of sertraline or quinacrine to CSDS mice, a rodent model of the disorder, moreover, did not improve depressive-like behaviors, differently from the treatment with ketamine, which displayed a more obvious antidepressant effect [55]. Although the positive effect of ketamine administration on depression features likely pivots on its main NMDAR antagonism pharmacodynamic mechanism, a Kir4.1-mediated effect on mice symptoms cannot be completely ruled out given the potential modulation of the channel induced by ketamine itself in astrocytes [46,91]. As stated by the same authors, the Kir4.1 channel may instead play a non-decisive role in this model of depression, although the effects of channel inhibitors would deserve to be investigated in long-term treatments, since previous studies showed that social avoidance in CSDS was reduced by chronic administration of fluoxetine and imipramine [92]. Pharmacological studies represent an additional and important piece of evidence supporting the role of Kir4.1 overexpression in depression [44,45,46,52,53]. A recent review on the effects of the novel antidepressant drugs on astrocytes also underlines the role of Kir4.1 upregulation in the pathogenesis of depression [41]: the authors hypothesized that increased Kir4.1 function might foster astrocyte-mediated glutamate uptake and consequently reduce the extracellular glutamate. This may lead to glutamatergic hypofunction, diminished BDNF levels, and loss of synaptic connectivity determining the depressive symptoms [41].

Taken together, the available literature is strongly unbalanced towards a role for Kir4.1 overexpression in the pathogenesis of depression, although some inconsistencies have emerged from the previous discussed literature. To deal with this aspect of the Kir4.1 metabolism, we can hypothesize the presence of conditions seen already in other Kir4.1-related conditions such as epilepsy, which is often seen in comorbidity with depression [93], where it has been documented either loss [94,95] or gain [96] of function *KCNJ10* variants. The two dysfunctional conditions of the Kir4.1 protein, respectively, lead to the Sesame/EAST syndrome [94,95] and the autism-epilepsy phenotype [96], both as a result of potassium siphoning alteration and possibly glutamatergic dysfunction. We cannot exclude that, similar to epilepsy conditions, depression can also be driven by diverse if not apparently opposite functional impairments of the Kir4.1 channel, namely a gain or loss of protein function. However, both dysfunctional conditions may definitely lead to glutamatergic dysfunction as a possible final common path of the channel’s impairment. The possible consequences of Kir4.1 downregulation on glutamate metabolism are relatively more straightforward, with respect to upregulation. Indeed, the loss of the channel function is expected to result in reduced astrocyte-siphoning with elevating extracellular potassium and glutamate, as it has been observed in cultured cortical astrocytes with knock-down of the channel [75] and in Kir4.1 knock-out conditional mice [76], and in neuronal hyperexcitability as it has been observed, e.g., in Noda epileptic rat [97]. Furthermore, the extracellular accumulation of glutamate could be responsible for excitotoxicity [75]. On the other hand, the functional consequences of channel upregulation still remain less obvious. Although one of the expected consequences of Kir4.1 upregulation primarily remains the increased glutamate uptake from the extracellular compartment, leading to glutamatergic hypofunction [41], the most robust findings that emerge from recent research in rodents strongly demonstrate the opposite. In particular, neuronal hyperpolarization due to the reduced K^+^ siphoning may lead to excessive bursting and glutamatergic hyperactivation, at least in specific neuronal networks or regions, for example, the LHb neurons investigated in the disease models from Cui and colleagues, eventually through Ca++ channels inactivation and NMDA receptors recruitment [37].

Expression studies of Kir4.1 in animal models revealed that the channel is weakly expressed during the first stages of the development, while it is strongly expressed in the adult stage [98]. This expression pattern correlates with the fluctuations of extracellular K^+^ at birth and its increased regulation in adulthood, when Kir4.1 expression saturates [99]. The progressive expression of Kir4.1 also correlates with glial differentiation [100]. The differences in the spatial and temporal expression of Kir4.1 during brain development in humans still remain largely unknown. However, we can hypothesize that they contribute to the diversity of disease manifestations that may result from channel dysfunction in different stages of human development, e.g., autism and/or epilepsy in infants or young children and depression in older patients [36,77,96].

5-HT: 5-hydroxytryptamine. 5-HTR: 5-hydroxytryptamine receptors. 5-HIAA: 5-hydroxyindoleacetic acid. VMAT: vesicular monoamine transporter. MAO-A: monoamine oxidase A. SERT: serotonin transporter. Kir4.1: inward rectifier potassium channel 4.1. AQP4: aquaporin 4. EAAT: excitatory amino acid transporter. Na^+^/K^+^ ATPase: sodium–potassium pump. GPCR: G protein-coupled receptor. BDNF: brain-derived neurotrophic factor. Glu: glutamate. VGLUT: vesicular glutamate transporter. GLUT: glucose transporter. GLN: glutamine. PAG: phosphate-activated glutaminase. AMPAR: α-amino-3-hydroxy-5-methyl-4-isoxazolepropionic acid receptor or AMPA receptor. NMDAR: N-methyl-D-aspartate receptor. mGluR: metabotropic glutamate receptor.

## 5. Conclusions

In conclusion, entangled neurobiological mechanisms likely underlie the heterogeneous spectrum of psychopathological conditions that manifest with depression as the main feature or as part of a clinical spectrum of mood/behavioral disorders. This clinical complexity leads clinicians to recognize several subtypes of depression probably reflecting the neurobiological heterogeneity described above. Furthermore, the seemingly conflicting nature of some literature data could simply reflect the polyhedral pathophysiology of this disorder, with Kir4.1 dysfunction possibly representing only one, although likely important, player for some, not for all, forms of depression. The comorbidity with other neurobiological conditions where Kir4.1 dysfunction belongs to the array of potential pathomechanisms, for example, epilepsy, may represent an additional important resource for research. Planning future genetic studies on clinical samples with comorbid conditions, will likely allow identifying those individuals where channel dysfunction can still have a relatively more central role compared to other mechanisms and eventually finding out closer genotype–phenotype correlations. This may in turn allow stratifying the population, within the heterogeneity of the disorder, through a “precision medicine” approach, a starting point for more tailored pharmacological treatments. Animal models of the disease will remain, however, the ideal platform for further clarification of the actual role of the Kir4.1-mediated astrocytic buffering and metabolism at the tripartite synapse.

## Figures and Tables

**Table 1:** Kir4.1 channel expression changes in depression.

Reference	Model	Brain Areas	Kir4.1 Expression
Medina et al., 2016 [38]	*Post-mortem* study on brain samples from patients with depression	Hippocampus	↓ Kir4.1
Cui et al., 2018 [37] and Yang et al., 2018 [54]	Rodent models of depression (cLH rat and LPS-treated rat)	Lateral Habenula	↑ Kir4.1
Xiong et al., 2019 [39]	*Post-mortem* study on brain samples from patients with depression	Parietal cortex	↑ Kir4.1
Xiong et al., 2019 [55]	Rodent models of depression (CSDS model)	Prefrontal cortex, nucleus accumbens septi and hippocampus	No change

cLH: congenitally learned helpless rat, LPS: lipopolysaccharide, CSDS: chronic social defeat stress.

**Table 2:** Effects of antidepressant drugs on Kir4.1 function/expression.

Reference	Drug	Model	Kir4.1 Expression/Function
Su et al., 2007 [44]	TCAs	HEK293T cells	↓ Kir4.1
Ohno et al., 2007 [45]	SSRIs	HEK293T cells	↓ Kir4.1
Furutani et al., 2009 [52]	TCAs and SSRIs	Chimeric and site directed mutants of Kir4.1 expressed in Xenopus Laevis oocytes and computational analyses of three-dimensional arrangements of the ligands.	↓ Kir4.1 interacting with channel pore residues
Kinboshi et al., 2017 [53]	SSRIs, TCAs, mianserin, and siRNA	Primary mouse astrocytes	↓ Kir4.1 and ↑ BDNF
Stenovec et al., 2020 [46]	Ketamine	Rat cortex astrocytes	↓ Kir4.1 reducing mobility of Kir4.1-carrying vesicles.
Song et al., 2019 [51] and Song et al., 2021 [50]	Fluoxetine	Normal adult rats	↓ Kir4.1 and ↑ BDNF

TCAs: tricyclic antidepressants; SSRIs: selective serotonin reuptake inhibitors, siRNA: RNA silencing; BDNF: brain derived neurotrophic factor.

## Supplementary Materials

The following are available online at https://www.mdpi.com/article/10.3390/cells10102628/s1. Supplementary Materials: The search strategy for the systematic review.

## References

[1] Hasin D.S., Sarvet A.L., Meyers J.L., Saha T.D., Ruan W.J., Stohl M., Grant B.F. (2018). Epidemiology of adult DSM-5 major depressive disorder and its specifiers in the United States. JAMA Psychiatry.

[2] Thapar A., Collishaw S., Pine D.S., Thapar A.K. (2012). Depression in adolescence. Lancet.

[3] American Psychiatric Association (2013). Diagnostic and Statistical Manual of Mental Disorders: DSM-5.

[4] Goldberg D. (2011). The heterogeneity of “major depression”. World Psychiatry.

[5] Delgado P.L. (2000). Depression: The case for a monoamine deficiency. J. Clin. Psychiatry.

[6] Hirschfeld R.M. (2000). History and evolution of the monoamine hypothesis of depression. J. Clin. Psychiatry.

[7] Cowen P.J. (2008). Serotonin and depression: Pathophysiological mechanism or marketing myth?. Trends Pharmacol. Sci..

[8] Coppen A. (1967). The Biochemistry of Affective Disorders. Br. J. Psychiatry.

[9] Cosci F., Chouinard G., Quevedo J., Carvalho A.F., Zarate C.A. (2019). The monoamine hypothesis of depression revisited: Could it mechanistically novel antidepressant strategies?. Neurobiology of Depression: Road to Novel Therapeutics.

[10] Freis E.D. (1954). Mental depression in hypertensive patients treated for long periods with large doses of reserpine. N. Engl. J. Med..

[11] Feighner J.P. (1990). Mechanism of Action of Antidepressant Medications. J. Clin. Psychiatry.

[12] American Psychiatric Association (2010). Practice Guideline for the Treatment of Patients with Major Depressive Disorder. https://psychiatryonline.org/pb/assets/raw/sitewide/practice_guidelines/guidelines/mdd.pdf.

[13] Fuller R.W. (1994). Uptake inhibitors increase extracellular serotonin concentration measured by brain microdialysis. Life Sci..

[14] American Academy of Pediatrics (2018). Guidelines for Adolescent Depression in Primary Care (GLAD-PC): Part II. Treatment and Ongoing Management..

[15] Al-Harbi K.S. (2012). Treatment-resistant depression: Therapeutic trends, challenges, and future directions. Patient Prefer Adherence.

[16] Björkholm C., Monteggia L.M. (2016). BDNF—A key transducer of antidepressant effects. Neuropharmacology.

[17] Liu B., Liu J., Wang M., Zhang Y., Li L. (2017). From Serotonin to Neuroplasticity: Evolvement of Theories for Major Depressive Disorder. Front. Cell Neurosci..

[18] Trullas R., Skolnick P. (1990). Functional antagonists at the NMDA receptor complex exhibit antidepressant actions. Eur. J. Pharmacol..

[19] Berman R.M., Cappiello A., Anand A., Oren D.A., Heninger G.R., Charney D.S., Krystal J.H. (2000). Antidepressant effects of ketamine in depressed patients. Biol. Psychiatry.

[20] Sanacora G., Treccani G., Popoli M. (2012). Towards a glutamate hypothesis of depression: An emerging frontier of neuropsychopharmacology for mood disorders. Neuropharmacology.

[21] Dwivedi Y. (2009). Brain-derived neurotrophic factor: Role in depression and suicide. Neuropsychiatr. Dis. Treat..

[22] Yu H., Chen Z.Y. (2011). The role of BDNF in depression on the basis of its location in the neural circuitry. Acta Pharmacol. Sin..

[23] Zhou X., Xiao Q., Xie L., Yang F., Wang L., Tu J. (2019). Astrocyte, a Promising Target for Mood Disorder Interventions. Front. Mol. Neurosci..

[24] Rajkowska G., Stockmeier C. (2013). Astrocyte Pathology in Major Depressive Disorder: Insights from Human Postmortem Brain Tissue. Curr. Drug Targets..

[25] Wang Q., Jie W., Yang J.L.J. (2017). An astroglial basis of major depressive disorder? An overview. Glia.

[26] Araque A., Sanzgiri R.P., Parpura V., Haydon P.G. (1999). Astrocyte-induced modulation of synaptic transmission. Can. J. Physiol. Pharmacol..

[27] Bellot-Saez A., Kékesi O., Morley J.W., Buskila Y. (2017). Astrocytic modulation of neuronal excitability through K+ spatial buffering. Neurosci. Biobehav. Rev..

[28] Molofsky A.V., Krenick R., Ullian E., Tsai H.H., Deneen B., Richardson W.D., Barres B.A., Rowitch D.H. (2012). Astrocytes and disease: A neurodevelopmental perspective. Genes Dev..

[29] Pekny M., Pekna M. (2016). Reactive gliosis in the pathogenesis of CNS diseases. Biochim. Biophys. Acta.

[30] Aida T., Yoshida J., Nomura M., Tanimura A., Iino Y., Soma M., Bai N., Ito Y., Cui W., Aizawa H. (2015). Astroglial glutamate transporter deficiency increases synaptic excitability and leads to pathological repetitive behaviors in mice. Neuropsychopharmacology.

[31] Altshuler L.L., Kupka R.W., Hellemann G., Frye M.A., Sugar C.A., McElroy S.L., Nolen W.A., Grunze H., Leverich G.S., Keck P.E. (2010). Gender and depressive symptoms in 711 patients with bipolar disorder evaluated prospectively in the Stanley Foundation Bipolar Treatment Outcome Network. Am. J. Psychiatry.

[32] Cobb J.A., O’Neill K., Milner J., Mahajan G.J., Lawrenca T.J., May W.L., Miguel-Hidalgo J., Rajkowska G. (2016). Density of GFAP-immunoreactive astrocytes is decreased in left hippocampi in major depressive disorder. Neuroscience.

[33] Rial D., Lemos C., Pinheiro H., Duarte J.M., Gonçalves F.Q., Real J.I., Prediger R.D., Gonçalves N., Gomes C.A., Canas P.M. (2016). Depression as a glial-based synaptic dysfunction. Front. Cell. Neurosci..

[34] Rubinow M.J., Mahajan G., May W., Overholser J.C., Jurjus G.J., Dieter L., Herbst N., Steffens D.C., Miguel-Hidalgo J.J., Rajkowska G. (2016). Basolateral amygdala volume and cell numbers in major depressive disorder: A postmortem stereological study. Brain Struct. Funct..

[35] Sanacora G. (2013). and Banasr, M. From pathophysiology to novel antidepressant drugs: Glial contributions to the pathology and treatment of mood disorders. Biol. Psychiatry.

[36] Nwaobi S.E., Cuddapah V.A., Patterson K.C., Randolph A.C., Olsen M.L. (2016). The role of glial-specific Kir4.1 in normal and pathological states of the CNS. Acta Neuropathol..

[37] Cui Y., Yang Y., Ni Z., Dong Y., Cai G., Foncelle A., Ma S., Sang K. (2018). Astroglial Kir4.1 in the lateral habenula drives neuronal bursts in depression. Nature.

[38] Medina A., Watson S.J., Bunney W., Myers R.M., Schatzberg A., Barchas J., Akil H., Thompson R.C. (2016). Evidence for alterations of the glial syncytial function in major depressive disorder. J. Psychiatr. Res..

[39] Xiong Z., Zhang K., Ren Q., Chang L., Chen J., Hashimoto K. (2019). Increased expression of inwardly rectifying Kir4.1 channel in the parietal cortex from patients with major depressive disorder. J. Affect. Disord..

[40] Ohno Y., Kinboshi M., Shimizu S. (2018). Inwardly rectifying potassium channel Kir4.1 as a novel modulator of BDNF expression in astrocytes. Int. J. Mol. Sci..

[41] Frizzo M.E., Ohno Y. (2021). Perisynaptic astrocytes as a potential target for novel antidepressant drugs. J. Pharmacol. Sci..

[42] Larsen B.R., MacAulay N. (2014). Kir4.1-mediated spatial buffering of K+: Experimental challenges in determination of its temporal and quantitative contribution to K+ clearance in the brain. Channels.

[43] Olsen M.L., Khakh B.S., Skatchkov S.N., Zhou M., Lee C.J., Rouach N. (2015). New insights on astrocyte ion channels: Critical for homeostasis and neuron-glia signaling. J. Neurosci..

[44] Su S., Ohno Y., Lossin C., Hibino H., Inanobe A., Kurachi Y. (2007). Inhibition of astroglial inwardly rectifying Kir4.1 channels by a tricyclic antidepressant, nortriptyline. J. Pharmacol. Exp. Ther..

[45] Ohno Y., Hibino H., Lossin C., Inanobe A., Kurachi Y. (2007). Inhibition of astroglial Kir4. 1 channels by selective serotonin reuptake inhibitors. Brain Res..

[46] Stenovec M., Božić M., Pirnat S., Zorec R. (2019). Astroglial Mechanisms of Ketamine Action Include Reduced Mobility of Kir4.1-Carrying Vesicles. Neurochem. Res..

[47] Zhang Z., Song Z., Shen F., Xie P., Wang J., Song Zhu A., Zhu G. (2021). Ginsenoside Rg1 Prevents PTSD-Like Behaviors in Mice Through Promoting Synaptic Proteins, Reducing Kir4.1 and TNF-α in the Hippocampus. Mol. Neurobiol..

[48] McIntyre R.S., Rosenblat J.D., Nemeroff C.B., Sanacora G., Murrough J.W., Berk M., Brietzke E., Dodd S., Gorwood P., Ho R. (2021). Synthesizing the Evidence for Ketamine and Esketamine in Treatment-Resistant Depression: An International Expert Opinion on the Available Evidence and Implementation. Am. J. Psychiatry.

[49] Liberati A., Altman D.G., Tetzlaff J., Mulrow C., Gøtzsche P.C., Ioannidis J.P.A., Clarke M., Devereaux P.J., Kleijnen J., Moher D. (2009). The PRISMA statement for reporting systematic reviews and meta-analyses of studies that evaluate healthcare interventions: Explanation and elaboration. BMJ.

[50] Song T., Chen W., Chen X., Zhang H., Zou Y., Wu H., Lin F., Ren L., Kang Y., Lei H. (2021). Repeated fluoxetine treatment induces transient and long-term astrocytic plasticity in the medial prefrontal cortex of normal adult rats. Prog. Neuro-Psychopharmacol. Biol. Psychiatry.

[51] Song T., Wu H., Li R., Xu H., Rao X., Gao L., Zou Y., Lei H. (2019). Repeated fluoxetine treatment induces long-lasting neurotrophic changes in the medial prefrontal cortex of adult rats. Behav. Brain Res..

[52] Furutani K., Ohno Y., Inanobe A., Hibino H., Kurachi Y. (2009). Mutational and in silico analyses for antidepressant block of astroglial inward-rectifier Kir4.1 channel. Mol. Pharmacol..

[53] Kinboshi M., Mukai T., Nagao Y., Matsuba Y., Tsuji Y., Tanaka S., Tokudome K., Shimizu S., Ito H., Ikeda A. (2017). Inhibition of inwardly rectifying potassium (Kir) 4.1 channels facilitates brain-derived neurotrophic factor (BDNF) expression in astrocytes. Front. Mol. Neurosci..

[54] Yang Y., Cui Y., Sang K., Dong Y., Ni Z., Ma S., Hu H. (2018). Ketamine blocks bursting in the lateral habenula to rapidly relieve depression. Nature.

[55] Xiong Z., Zhang K., Ishima T., Ren Q., Ma M., Pu Y., Chang L., Chen J., Hashimoto K. (2019). Lack of rapid antidepressant effects of Kir4.1 channel inhibitors in a chronic social defeat stress model: Comparison with (R)-ketamine. Pharmacol. Biochem. Behav..

[56] Toyoda H., Li X.Y., Wu L.J., Zhao M.G., Descalzi G., Chen T., Koga K., Zhuo M. (2011). Interplay of amygdala and cingulate plasticity in emotional fear. Neural Plast..

[57] Anand K., Dhikav V. (2012). Hippocampus in health and disease: An overview. Ann. Indian Acad. Neurol..

[58] Campbell S., MacQueen G. (2004). The role of the hippocampus in the pathophysiology of major depression. J. Psychiatry Neurosci..

[59] Pandya M., Altinay M., Malone D.A., Anand A. (2012). Where in the brain is depression?. Curr. Psychiatry Rep..

[60] Strohschein S., Hüttmann K., Gabriel S., Binder D.K., Heinemann U., Steinhäuser C. (2011). Impact of aquaporin-4 channels on K + buffering and gap junction coupling in the hippocampus. Glia.

[61] Proulx C.D., Hikosaka O., Malinow R. (2014). Reward processing by the lateral habenula in normal and depressive behaviors. Nat. Neurosci..

[62] Yang Y., Wang H., Hu J., Hu H. (2018). Lateral habenula in the pathophysiology of depression. Curr. Opin. Neurobiol..

[63] Browne C.A., Hammack R., Lucki I. (2018). Dysregulation of the Lateral Habenula in Major Depressive Disorder. Front. Synaptic Neurosci..

[64] Hammen C. (2005). Stress and depression. Annu. Rev. Clin. Psychol..

[65] George M.S., Ketter T.A., Post R.M. (1994). Prefrontal cortex dysfunction in clinical depression. Depression.

[66] Liu W., Ge T., Leng Y., Pan Z., Fan J., Yang W., Cui R. (2017). The Role of Neural Plasticity in Depression: From Hippocampus to Prefrontal Cortex. Neural Plast..

[67] Heshmati M., Russo S.J. (2015). Anhedonia and the Brain Reward Circuitry in Depression. Curr. Behav. Neurosci. Rep..

[68] Pizzagalli D.A., Holmes A.J., Dillon D.G., Goetz E.L., Birk J.L., Bogdan R., Dougherty D.D., Iosifescu D.V., Rauch S.L., Fava M. (2009). Reduced Caudate and Nucleus Accumbens Response to Rewards in Unmedicated Subjects with Major Depressive Disorder. Am. J. Psychiatry.

[69] MacQueen G., Frodl T. (2011). The hippocampus in major depression: Evidence for the convergence of the bench and bedside in psychiatric research. Mol. Psychiatry.

[70] Groves J.O. (2007). Is it time to reassess the BDNF hypothesis of depression?. Mol. Psychiatry.

[71] Binder D.K., Croll S.D., Gall C.M., Scharfman H.E. (2001). BDNF and epilepsy: Too much of a good thing?. Trends Neurosci..

[72] Iughetti L., Lucaccioni L., Fugetto F., Predieri B., Berardi A., Ferrari F. (2018). Brain-derived neurotrophic factor and epilepsy: A systematic review. Neuropeptides.

[73] Treadway M.T., Waskom M.L., Dillon D.G., Holmes A.J., Park M.T.M., Chakravarty M.M., Dutra S.J., Polli F.E., Iosifescu D.V., Fava M. (2015). Illness progression, recent stress, and morphometry of hippocampal subfields and medial prefrontal cortex in major depression. Biol. Psychiatry.

[74] Guo F., Zhang Q., Zhang B., Fu Z., Wu B., Huang C., Li Y. (2014). Burst-firing patterns in the prefrontal cortex underlying the neuronal mechanisms of depression probed by antidepressants. Eur. J. Neurosci..

[75] Kucheryavykh Y.V., Kucheryavykh L.Y., Nichols C.G., Maldonado H.M., Baksi K., Reichenbach A., Skatchkov S.N., Eaton M.J. (2007). Downregulation of Kir4.1 inward rectifying potassium channel subunits by RNAi impairs potassium transfer and glutamate uptake by cultured cortical astrocytes. Glia.

[76] Djukic B., Casper K.B., Philpot B.D., Chin L.S., McCarthy K.D. (2007). Conditional knock-out of Kir4.1 leads to glial membrane depolarization, inhibition of potassium and glutamate uptake, and enhanced short-term synaptic potentiation. J. Neurosci..

[77] Sicca F., Ambrosini E., Marchese M., Sforna L., Servettini I., Valvo G., Brignone M.S., Lanciotti A., Moro F., Grottesi A. (2016). Gain-of-function defects of astrocytic Kir4.1 channels in children with autism spectrum disorders and epilepsy. Sci. Rep..

[78] Mathews D.C., Henter I.D., Zarate C.A. (2012). Targeting the Glutamatergic System to Treat Major Depressive Disorder. Drugs.

[79] Rodrigues F.T.S., de Souza M.R.M., Lima C.N.C., da Silva F.E.R., Costa D.V.D.S., Dos Santos C.C., Miyajima F., de Sousa F.C.F., Vasconcelos S.M.M., Barichello T. (2018). Major depression model induced by repeated and intermittent lipopolysaccharide administration: Long-lasting behavioral, neuroimmune and neuroprogressive alterations. J. Psychiatr. Res..

[80] Jiang B., Xiong Z., Yang J., Wang W., Wang Y., Hu Z.L., Wang F., Chen J.G. (2012). Antidepressant-like effects of ginsenoside Rg1 are due to activation of the BDNF signalling pathway and neurogenesis in the hippocampus. Br. J. Pharmacol..

[81] Kim Y., Cho S.H. (2021). The effect of ginsenosides on depression in preclinical studies: A systematic review and meta-analysis. J. Ginseng Res..

[82] Wotton C.A., Cross C.D., Bekar L.K. (2020). Serotonin, norepinephrine and acetylcholine differentially affect astrocytic potassium clearance to modulate somatosensory signaling in male mice. J. Neurosci Res..

[83] Puissant M.M., Mouradian G.C., Liu P., Hodges M.R. (2017). Identifying Candidate Genes that Underlie Cellular pH Sensitivity in Serotonin Neurons Using Transcriptomics: A Potential Role for Kir5.1 Channels. Front. Cell. Neurosci..

[84] De Carvalho D., Patrone L.G., Taxini C.L., Biancardi V., Vicente M.C., Gargaglioni L.H. (2014). Neurochemical and electrical modulation of the locus coeruleus: Contribution to CO2drive to breathe. Front. Physiol..

[85] Kim M.A., Lee H.S., Lee B.Y., Waterhouse B.D. (2004). Reciprocal connections between subdivisions of the dorsal raphe and the nuclear core of the locus coeruleus in the rat. Brain Res..

[86] Benfenati V., Caprini M., Nobile M., Rapisarda C., Ferroni S. (2006). Guanosine promotes the up-regulation of inward rectifier potassium current mediated by Kir4.1 in cultured rat cortical astrocytes. J. Neurochem..

[87] Hibino H., Higashi-Shingai K., Fujita A., Iwai K., Ishii M., Kurachi Y. (2004). Expression of an inwardly rectifying K+ channel, Kir5.1, in specific types of fibrocytes in the cochlear lateral wall suggests its functional importance in the establishment of endocochlear potential. Eur. J. Neurosci..

[88] D’Adamo M.C., Shang L., Imbrici P., Brown S.D., Pessia M., Tucker S.J. (2011). Genetic inactivation of Kcnj16 identifies Kir5.1 as an important determinant of neuronal PCO2/pH sensitivity. J. Biol. Chem..

[89] Martinowich K., Manji H., Lu B. (2007). New insights into BDNF function in depression and anxiety. Nat. Neurosci..

[90] Yang T., Nie Z., Shu H., Kuang Y., Chen X., Cheng J., Yu S., Liu H. (2020). The Role of BDNF on Neural Plasticity in Depression. Front. Cell. Neurosci..

[91] Stenovec M., Li B., Verkhratsky A., Zorec R. (2020). Astrocytes in rapid ketamine antidepressant action. Neuropharmacology.

[92] Tsankova N.M., Berton O., Renthal W., Kumar A., Neve R.L., Nestler E.J. (2006). Sustained hippocampal chromatin regulation in a mouse model of depression and antidepressant action. Nat. Neurosci..

[93] Salpekar J.A., Mula M. (2019). Common psychiatric comorbidities in epilepsy: How big of a problem is it?. Epilepsy Behav..

[94] Scholl U.I., Choi M., Liu T., Ramaekers V.T., Häusler M.G., Grimmer J., Tobe S.W., Farhi A., Nelson-Williams C., Lifton R.P. (2009). Seizures, sensorineural deafness, ataxia, mental retardation, and electrolyte imbalance (SeSAME syndrome) caused by mutations in KCNJ10. Proc. Natl. Acad. Sci. USA.

[95] Bockenhauer D., Feather S., Stanescu H.C., Bandulik S., Zdebik A.A., Reichold M., Tobin J., Lieberer E., Sterner C., Landoure G. (2009). Epilepsy, ataxia, sensorineural deafness, tubulopathy, and KCNJ10 mutations. N. Engl. J. Med..

[96] Sicca F., Imbrici P., D’Adamo M.C., Moro F., Bonatti F., Brovedani P., Grottesi A., Guerrini R., Masi G., Santorelli F.M. (2011). Autism with Seizures and Intellectual Disability: Possible Causative Role of Gain-of-function of the Inwardly-Rectifying K + Channel Kir4.1. Neurobiol. Dis..

[97] Harada Y., Nagao Y., Shimizu S., Serikawa T., Terada R., Fujimoto M., Okuda A., Mukai T., Sasa M., Kurachi Y. (2013). Expressional analysis of inwardly rectifying Kir4.1 channels in Noda epileptic rat (NER). Brain Res..

[98] Olsen M.L., Higashimori H., Campbell S.L., Hablitz J.J., Sontheimer H. (2006). Functional expression of K(ir)4.1 channels in spinal cord astrocytes. Glia.

[99] Connors B.W., Ransom B.R., Kunis D.M., Gutnick M.J. (1982). Activity-dependent K+ accumulation in the developing rat optic nerve. Science.

[100] Moroni R.F., Inverardi F., Regondi M.C., Pennacchio P., Frassoni C. (2015). Developmental expression of Kir4.1 in astrocytes and oligodendrocytes of rat somatosensory cortex and hippocampus. Int. J. Dev. Neurosci..

