# Supplementary material for: Kir4.1 Dysfunction in the Pathophysiology of Depression: A Systematic Review
Source: Cells. 2021 Oct 1;10(10):2628. doi: 10.3390/cells10102628 (PMC8534194; doi:10.3390/cells10102628)
Supplement: Supplementary file 1 [file cells-10-02628-s001.zip › cells-1378713-supplementary.pdf]

Supplementary Materials The search strategy for the systematic review.

**Identify Key Concept**	
Component 1	depress* OR antidepre*
Component 2	Kir4.1 OR inward rectifying 4.1 OR KCNJ10
**Database selection**	
PubMed, Scopus, Web of Science	
**Combine Search Terms with Boolean Operators**	
Pubmed	(depress* OR antidepre*) AND (Kir4.1 OR inward rectifying 4.1 OR KCNJ10)
Scopus	TITLE-ABS-KEY ((depress* OR antidepre*) AND (Kir4.1 OR inward rectifying 4.1 OR KCNJ10))
Web of science	((depress* OR antidepre*) AND (Kir4.1 OR inward rectifying 4.1 OR KCNJ10))
**Papers Identification**	
Pubmed	n = 29
Scopus	n = 8
Web of science	n = 43
Others sources: List of papers' references	n = 2
**Selection Criteria**	
**Inclusion Criteria**	**Exclusion Criteria**
1. Studies reported on the relationship between the astrocytic Kir4.1 channel and antidepressant drugs. The antidepressant drugs that were considered for the inclusion of the studies in our systematic review were those recommended in the treatment guidelines for adults (Available from: https://psychiatryonline.org/pb/assets/raw/sitewide/practice_guidelines/guidelines/mdd.pdf.) and young people (Available from: https://pediatrics.aappublications.org/content/pediatrics/early/2018/02/22/peds.2017-4082.full.pdf.) ∄ (i.e., SSRIs, SNRIs, TCAs, monoamine oxidase inhibitors, atypical antidepressants), and the more recently FDA (Food and Drug Administration) approved drug for resistant depression ketamine (PMID: 33726522).	(a) duplicates<br>(b) studies not concerning the aim of the paper<br>(c) studies focusing on drugs that were not approved for depression treatment in humans or with alleged antidepressant effects only based on limited evidence in animal models<br>(d) articles written in a language other than English, French, or Italian
2. Studies reported on the relationship between the astrocytic Kir4.1 channel and depression	(e) reviews, meeting abstracts and editorial comments
**Duplicate removal**	
80–30 = 50 papers	
**Papers screened**	
N = 50	
**Records included**	**Records excluded**
N = 12	Different topic (n = 20)
	Review article (n = 10)
	Meeting abstract (n = 4)
	Editorial comments (n = 2)
	Drugs not approved for depression (n = 2)

**Papers Selection of Articles According to Inclusion and Exclusion Criteria**
**Included Studies**
Cui, Y.; Yang, Y.; Ni, Z.; Dong, Y.; Cai, G.; Foncelle, A.; Ma, S.; Sang, K. Astroglial Kir4.1 in the lateral habenula drives neuronal bursts in depression. *Nature*. **2018**, *554*, 323-327.

Medina, A.; Watson, S.J.; Bunney, W.; Myers, R.M.; Schatzberg, A.; Barchas, J.; Akil, H.; Thompson, R.C. Evidence for alterations of the glial syncytial function in major depressive disorder. *J. Psychiatr. Res*. **2016**, *72*, 15-21.	
Xiong, Z.; Zhang, K.; Ren, Q.; Chang, L.; Chen, J.; Hashimoto, K. Increased expression of inwardly rectifying Kir4.1 channel in the parietal cortex from patients with major depressive disorder. *J. Affect. Disord*. **2019**, *245*, 265–269.	
Su, S.; Ohno, Y.; Lossin, C.; Hibino, H.; Inanobe, A.; Kurachi, Y. Inhibition of astroglial inwardly rectifying Kir4.1 channels by a tricyclic antidepressant, nortriptyline. *J. Pharmacol. Exp. Ther*. **2007**, *320*, 573–580.	
Ohno, Y.; Hibino, H.; Lossin, C.; Inanobe, A.; Kurachi, Y. Inhibition of astroglial Kir4.1 channels by selective serotonin reuptake inhibitors. *Brain Res*. **2007**, *1178*, 44-51.	
Stenovec, M.; Božić, M.; Pirnat, S.; Zorec, R. Astroglial Mechanisms of Ketamine Action Include Reduced Mobility of Kir4.1-Carrying Vesicles. *Neurochem. Res*. **2019**, *45*, 109-121.	
Song, T.; Chen, W.; Chen, X.; Zhang, H.; Zou,Y.; Wu, H.; Lin, F.; Ren, L.; Kang, Y.; Lei, H. Repeated fluoxetine treatment induces transient and long-term astrocytic plasticity in the medial prefrontal cortex of normal adult rats. *Prog. Neuro-Psychopharmacology Biol. Psychiatry*. **2021**, *107*, 110252.	
Song, T.; Wu, H.; Li, R.; Xu, H.; Rao, X.; Gao, L.; Zou, Y.; Lei, H. Repeated fluoxetine treatment induces long-lasting neurotrophic changes in the medial prefrontal cortex of adult rats. *Behav. Brain Res*. **2019**, *365*, 114–124.	
Furutani, K.; Ohno, Y.; Inanobe, A.; Hibino, H.; Kurachi, Y. Mutational and in silico analyses for antidepressant block of astroglial inward-rectifier Kir4.1 channel. *Mol. Pharmacol*. **2009**, *75*, 1287–1295.	
Kinboshi, M.; Mukai, T.; Nagao, Y.; Matsuba, Y.; Tsuji, Y.; Tanaka, S.; Tokudome, K.; Shimizu, S.; Ito, H.; Ikeda, A.; Inanobe, A.; Kurachi, Y.; Inoue, S.; Ohno, Y. Inhibition of inwardly rectifying potassium (Kir) 4.1 channels facilitates brain-derived neurotrophic factor (BDNF) expression in astrocytes. *Front. Mol. Neurosci*. **2017**, *10*, 408.	
Yang, Y.; Cui, Y.; Sang, K.; Dong, Y.; Ni, Z.; Ma, S.; Hu, H. Ketamine blocks bursting in the lateral habenula to rapidly relieve depression. *Nature*. **2018**, *554*, 317-322.	
Xiong, Z.; Zhang, K.; Ishima, T.; Ren, Q.; Ma, M.; Pu, Y.; Chang, L.; Chen, J.; Hashimoto, K. Lack of rapid antidepressant effects of Kir4.1 channel inhibitors in a chronic social defeat stress model: Comparison with (R)-ketamine. *Pharmacol. Biochem. Behav*. **2019**, *176*, 57–62.	
**Excluded Studies**	
Behzad E, Ghabaee M, Bigdeli M R, Noorbakhsh F, Gorji A, et al. Effect of Nortriptyline on Spreading Depolarization, Arch Neurosci. Online ahead of Print ; 7(3):e102102, doi:10.5812/ans.102102.	Different topic
Benfenati V, Caprini M, Nobile M, Rapisarda C, Ferroni S. Guanosine promotes the up-regulation of inward rectifier potassium current mediated by Kir4.1 in cultured rat cortical astrocytes. J Neurochem. 2006 Jul;98(2):430-45, doi:10.1111/j.1471-4159.2006.03877.x. PMID: 16805837.	Drugs not approved for depression
Chen C, Chen H, Xu C, Zhong Y, Shen X. Role of interleukin-1β in hypoxia-induced depression of glutamate uptake in retinal Müller cells. Graefes Arch Clin Exp Ophthalmol. 2014 Jan;252(1):51-8, doi:10.1007/s00417-013-2516-z. Epub 2013 Nov 12. PMID: 24218043.	Different topic
Colbourn, Robert et al. "Rapid volume pulsation of the extracellular space coincides with epileptiform activity in mice and depends on the NBCe1 transporter." *The Journal of Physiology* 599 (2021): n. pag.	Different topic
Frizzo M. E. (2017). Can a Selective Serotonin Reuptake Inhibitor Act as a Glutamatergic Modulator?. *Current therapeutic research, clinical and experimental*, *87*, 9–12. https://doi.org/10.1016/j.curtheres.2017.07.001	Review
Frizzo ME. The Effect of Glutamatergic Modulators on Extracellular Glutamate: How Does this Information Contribute to the Discovery of Novel Antidepressants? Curr Ther Res Clin Exp. 2019 Sep 10;91:25-32, doi:10.1016/j.curtheres.2019.100566. PMID: 31871505; PMCID: PMC6911922.	Review
Fujita, A., Horio, Y., Higashi, K., Mouri, T., Hata, F., Takeguchi, N., & Kurachi, Y. (2002). Specific localization of an inwardly rectifying K(+) channel, Kir4.1, at the apical membrane of rat gastric parietal cells; its possible involvement in K(+) recycling for the H(+)-K(+)-pump. *The Journal of physiology*, *540*(Pt 1), 85–92. https://doi.org/10.1113/jphysiol.2001.013439	Different topic
Furutani, K., Inanobe, A., & Kurachi, Y. (2009). The Structural Basis for Antidepressants Block Being Confined to Kir4.1. *Biophysical Journal*, *96*, 4-5.	Meeting Abstract

Hashimoto K. Rapid-acting antidepressant ketamine, its metabolites and other candidates: A historical overview and future perspective. Psychiatry Clin Neurosci. 2019 Oct;73(10):613-627, doi:10.1111/pcn.12902. Epub 2019 Jul 11. PMID: 31215725; PMCID: PMC6851782.	Review
Hertz L, Chen Y. Importance of astrocytes for potassium ion (K+) homeostasis in brain and glial effects of K+ and its transporters on learning. Neurosci Biobehav Rev. 2016 Dec;71:484-505, doi:10.1016/j.neubiorev.2016.09.018. Epub 2016 Sep 28. PMID: 27693230.	Review
Hibino H, Horio Y, Inanobe A, Doi K, Ito M, Yamada M, Gotow T, Uchiyama Y, Kawamura M, Kubo T, Kurachi Y. An ATP-dependent inwardly rectifying potassium channel, KAB-2 (Kir4. 1), in cochlear stria vascularis of inner ear: its specific subcellular localization and correlation with the formation of endocochlear potential. J Neurosci. 1997 Jun 15;17(12):4711-21, doi:10.1523/JNEUROSCI.17-12-04711.1997. PMID: 9169531; PMCID: PMC6573344.	Different topic
Ji, M., Miao, Y., Dong, L. D., Chen, J., Mo, X. F., Jiang, S. X., Sun, X. H., Yang, X. L., & Wang, Z. (2012). Group I mGluR-mediated inhibition of Kir channels contributes to retinal Müller cell gliosis in a rat chronic ocular hypertension model. *The Journal of neuroscience : the official journal of the Society for Neuroscience*, *32*(37), 12744–12755. https://doi.org/10.1523/JNEUROSCI.1291-12.2012	Different topic
Johnen A, Bürkner PC, Landmeyer NC, Ambrosius B, Calabrese P, Motte J, Hessler N, Antony G, König IR, Klotz L, Hoshi MM, Aly L, Groppa S, Luessi F, Paul F, Tackenberg B, Bergh FT, Kümpfel T, Tumani H, Stangel M, Weber F, Bayas A, Wildemann B, Heesen C, Zettl UK, Zipp F, Hemmer B, Meuth SG, Gold R, Wiendl H, Salmen A; German Competence Network Multiple Sclerosis (KKNMS). Can we predict cognitive decline after initial diagnosis of multiple sclerosis? Results from the German National early MS cohort (KKNMS). J Neurol. 2019 Feb;266(2):386-397, doi:10.1007/s00415-018-9142-y. Epub 2018 Dec 4. PMID: 30515631; PMCID: PMC6373354.	Different topic
Kang, S (Kang, Seema). Astroglial Kir4.1 in depression. Lancet psychiatry. Volume5, Issue4. Page301-301. Published APR 2018.	Editorial comments
Kaprielian, R., Wickenden, A.D., Kassiri, Z., Parker, T.G., Liu, P., & Backx, P.H. (1999). Relationship between K+ channel down-regulation and [Ca2+]i in rat ventricular myocytes following myocardial infarction. *The Journal of Physiology, 517*.	Different topic
Kim D, Cheong E, Shin HS. Overcoming Depression by Inhibition of Neural Burst Firing. Neuron. 2018 Jun 6;98(5):878-879, doi:10.1016/j.neuron.2018.05.032. PMID: 29879390.	Review
Lu DC, Zhang H, Zador Z, Verkman AS. Impaired olfaction in mice lacking aquaporin-4 water channels. FASEB J. 2008 Sep;22(9):3216-23, doi:10.1096/fj.07-104836. Epub 2008 May 29. PMID: 18511552; PMCID: PMC2518258.	Different topic
Marmolejo-Murillo LG, Aréchiga-Figueroa IA, Moreno-Galindo EG, Navarro-Polanco RA, Rodríguez-Menchaca AA, Cui M, Sánchez-Chapula JA, Ferrer T. Chloroquine blocks the Kir4.1 channels by an open-pore blocking mechanism. Eur J Pharmacol. 2017 Apr 5;800:40-47, doi:10.1016/j.ejphar.2017.02.024. Epub 2017 Feb 20. PMID: 28216048.	Different topic
Ohno Y. Astrocytic Kir4.1 potassium channels as a novel therapeutic target for epilepsy and mood disorders. Neural Regen Res. 2018 Apr;13(4):651-652, doi:10.4103/1673-5374.230355. PMID: 29722316; PMCID: PMC5950674.	Editorial comments
Padmawar P, Yao X, Bloch O, Manley GT, Verkman AS. K+ waves in brain cortex visualized using a long-wavelength K+-sensing fluorescent indicator. Nat Methods. 2005 Nov;2(11):825-7, doi:10.1038/nmeth801. PMID: 16278651.	Different topic
Peng L, Verkhratsky A, Gu L, Li B. Targeting astrocytes in major depression. Expert Rev Neurother. 2015;15(11):1299-306, doi:10.1586/14737175.2015.1095094. Epub 2015 Oct 15. PMID: 26471936.	Review
Raphemot R, Kadakia RJ, Olsen ML, Banerjee S, Days E, Smith SS, Weaver CD, Denton JS. Development and validation of fluorescence-based and automated patch clamp-based functional assays for the inward rectifier potassium channel Kir4.1. Assay Drug Dev Technol. 2013 Nov-Dec;11(9-10):532-43, doi:10.1089/adt.2013.544. Epub 2013 Nov 22. PMID: 24266659; PMCID: PMC3870600.	Different topic

Rodríguez-Menchaca, A.A., Aréchiga-Figueroa, I.A. & Sánchez-Chapula, J.A. The molecular basis of chloroethylclonidine block of inward rectifier (Kir2.1 and Kir4.1) K+ channels. *Pharmacol. Rep* **68,** 383–389 (2016). https://doi.org/10.1016/j.pharep.2015.10.005	Different topic
Schnell C, Janc OA, Kempkes B, Callis CA, Flügge G, Hülsmann S, Müller M. Restraint Stress Intensifies Interstitial K(+) Accumulation during Severe Hypoxia. Front Pharmacol. 2012 Mar 28;3:53, doi:10.3389/fphar.2012.00053. PMID: 22470344; PMCID: PMC3314232.	Different topic
Sibille, J., Dao Duc, K., Holcman, D., & Rouach, N. (2015). The neuroglial potassium cycle during neurotransmission: role of Kir4.1 channels. *PLoS computational biology*, *11*(3), e1004137. https://doi.org/10.1371/journal.pcbi.1004137	Different topic
Skatchkov SN, Eaton MJ, Shuba YM, Kucheryavykh YV, Derst C, Veh RW, Wurm A, Iandiev I, Pannicke T, Bringmann A, Reichenbach A. Tandem-pore domain potassium channels are functionally expressed in retinal (Müller) glial cells. Glia. 2006 Feb;53(3):266-76, doi:10.1002/glia.20280. PMID: 16265669.	Different topic
Stecyk JA, Paajanen V, Farrell AP, Vornanen M. Effect of temperature and prolonged anoxia exposure on electrophysiological properties of the turtle (Trachemys scripta) heart. Am J Physiol Regul Integr Comp Physiol. 2007 Jul;293(1):R421-37, doi:10.1152/ajpregu.00096.2007. Epub 2007 Apr 18. PMID: 17442785.	Different topic
Stenovec M, Bozic M, Pirnat S, Lasic E, Zorec R. Unraveling the Role of Astroglia in the Mechanism of Antidepressant Action of Ketamine. Neuroendocrinology, Volume111, IssueSUPPL 1, Page39-40, Supplement1. Published MAR 2021.	Meeting Abstract
Stenovec M, Li B, Verkhratsky A, Zorec R. Astrocytes in rapid ketamine antidepressant action. Neuropharmacology. 2020 Aug 15;173:108158, doi:10.1016/j.neuropharm.2020.108158. Epub 2020 May 25. PMID: 32464133.	Review
Weaver CD, Denton JS. Next-generation inward rectifier potassium channel modulators: discovery and molecular pharmacology. Am J Physiol Cell Physiol. 2021 Jun 1;320(6):C1125-C1140, doi:10.1152/ajpcell.00548.2020. Epub 2021 Apr 7. PMID: 33826405; PMCID: PMC8285633.	Review
Wenker IC, Kréneisz O, Nishiyama A, Mulkey DK. Astrocytes in the retrotrapezoid nucleus sense H+ by inhibition of a Kir4.1-Kir5.1-like current and may contribute to chemoreception by a purinergic mechanism. J Neurophysiol. 2010 Dec;104(6):3042-52, doi:10.1152/jn.00544.2010. Epub 2010 Oct 6. PMID: 20926613; PMCID: PMC3007661.	Different topic
Wu SN, Huang YM, Kao CA, Chen BS, Lo YC. Investigations on contribution of glial inwardly-rectifying K(+) current to membrane potential and ion flux: an experimental and theoretical study. Kaohsiung J Med Sci. 2015 Jan;31(1):9-17, doi:10.1016/j.kjms.2014.10.006. Epub 2014 Nov 22. PMID: 25600915.	Different topic
Zhang X, Su J, Cui N, Gai H, Wu Z, Jiang C. The disruption of central CO2 chemosensitivity in a mouse model of Rett syndrome. Am J Physiol Cell Physiol. 2011 Sep;301(3):C729-38, doi:10.1152/ajpcell.00334.2010. Epub 2011 Feb 9. PMID: 21307341; PMCID: PMC3174562.	Different topic
Zhang Z, Song Z, Shen F, Xie P, Wang J, Zhu AS, Zhu G. Ginsenoside Rg1 Prevents PTSD-Like Behaviors in Mice Through Promoting Synaptic Proteins, Reducing Kir4.1 and TNF-α in the Hippocampus. Mol Neurobiol. 2021 Apr;58(4):1550-1563, doi:10.1007/s12035-020-02213-9. Epub 2020 Nov 19. PMID: 33215390; PMCID: PMC7676862.	Drugs not approved for depression
Frizzo ME, Ohno Y. Perisynaptic astrocytes as a potential target for novel antidepressant drugs. J Pharmacol Sci. 2021 Jan;145(1):60-68, doi:10.1016/j.jphs.2020.11.002. Epub 2020 Nov 11. PMID: 33357781.	Review
Furutani K, Ohno Y, Hibino H, Inanobe A, Kurachi Y. Antidepressants block astroglial Kir4.1 channel by binding in the central cavity. Journal of pharmacological sciences. Volume106. Page134P-134P. Supplement1. Published2008. LocationYokohama, JAPAN. DateMAR 17-19, 2008. SponsorJapanese Pharmacol Soc	Meeting Abstract
Ohno, Y. Molecular mechanisms for the block of Kir4.1 channels by antidepressants. ISSN: 1347-8613. Journal of pharmacological sciences, Vol.106, p.133-133	Meeting Abstract
